# The Community Assessment to Inform Rapid Response (CAIRR): A Novel Qualitative Data Collection and Analytic Process to Facilitate Hyperlocal COVID-19 Emergency Response Operations in New York City

**DOI:** 10.1017/dmp.2022.135

**Published:** 2022-05-30

**Authors:** Madhury Ray, Rachel Dannefer, Jennifer Pierre, Lauren J Shiman, Hannah L Helmy, Shelby R Boyle, Jae Eun M Chang, Alyssa Creighton, Maria A Soto, Jacqlene Moran

**Affiliations:** New York City Department of Health and Mental Hygiene, New York, NY, USA

**Keywords:** hyperlocal, disaster response, health equity, COVID-19 incident command system

## Abstract

All disasters are local but implementing a hyperlocal response in the midst of a public health emergency is challenging. The availability of neighborhood-level qualitative data that are both timely and relevant to evolving objectives and operations is a limiting factor. In 2020, the New York City Department of Health and Mental Hygiene (NYC DOHMH) responded to the COVID-19 emergency using a novel, hyperlocal approach. Key to the implementation of this approach was the creation of the Community Assessment to Inform Rapid Response (CAIRR), a process for rapid collection and analysis of neighborhood-specific, objective-focused, qualitative data to inform tailored response operations. This paper describes the process of developing the CAIRR and its contribution to the NYC DOHMH’s hyperlocal response in order to guide other jurisdictions seeking to employ a hyperlocal approach in future disaster responses.

The first case of COVID-19 in New York City (NYC) was confirmed on February 29, 2020, though data suggest that community transmission of SARS-CoV-2 was already widespread at that point.^1^ By the end of March 2020, NYC had become the global epicenter of the COVID-19 pandemic.^2^ Significant racial and ethnic disparities in SARS-CoV-2 transmission as well as COVID-19-related morbidity and mortality soon became evident in NYC, including a disproportionate impact among Black, Latinx, Asian, and Indigenous individuals relative to White individuals.^2–6^ This pattern of disparity by race in NYC is consistent with population-level observations of racial inequity in chronic health conditions and life expectancy,^7^ as well as in the impacts of other public health emergencies.^8–10^ Numerous studies have described the mechanisms of structural racism underlying and amplifying these inequities, notably that generations of practices and policies have resulted in the ongoing exclusion of communities of color from resources and conditions that promote health and increase resilience.^11–15^

In NYC, many of these policies targeted neighborhoods based explicitly on their racial demographics, creating health inequities that are both racial and geographic.^16–18^ In the context of the early COVID-19 emergency, those geographic disparities in health translated to persistently differential rates of SARS-CoV-2 transmission and COVID-19 related morbidity and mortality across different NYC neighborhoods, despite the implementation of citywide response operations (See Figure 1).^3^ At the same time, neighborhood-level work by the NYC Department of Health and Mental Hygiene (NYC DOHMH) suggested that as the impacts of COVID-19 differed by neighborhood, so too did the constellation of public health barriers and resources shaping SARS-CoV-2 transmission in each neighborhood. The persistence of geographic disparities despite multilayered non-pharmaceutical interventions at the city level uncovered an urgent need for a novel, place-based approach to emergency response, tailored to the unique public health barriers, and resources in neighborhoods disproportionately impacted by the pandemic.

**Figure 1.**
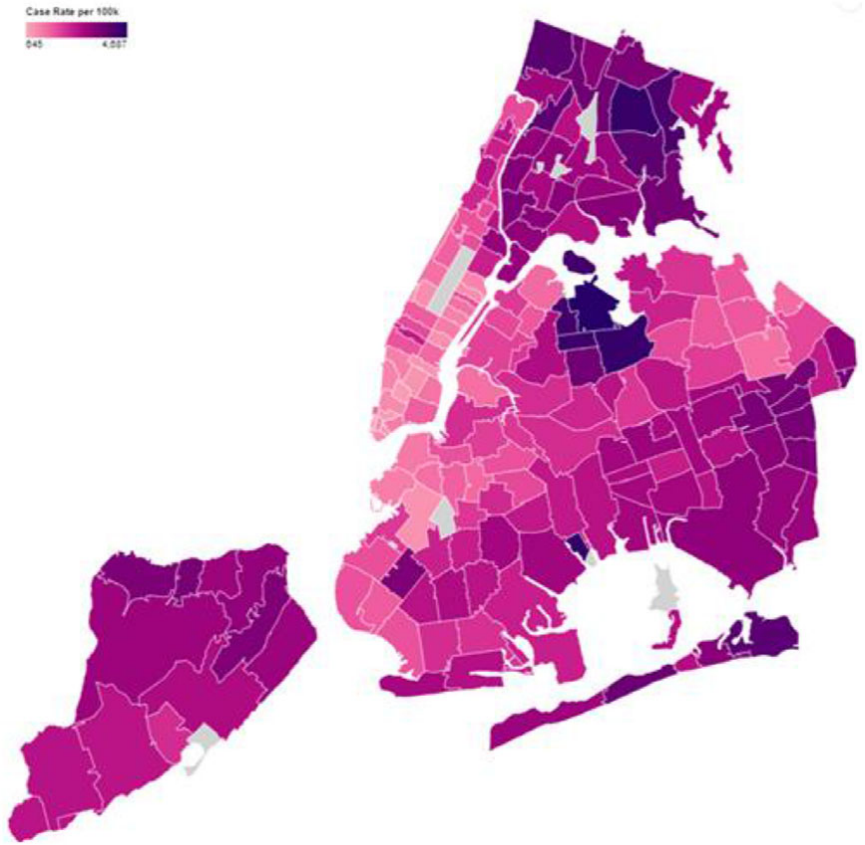
Cumulative confirmed SARS-CoV-2 case rates by neighborhood in NYC (July 2020). The map illustrates the disparities in cumulative SARS-CoV-2 PCR postive case rates by neighborhood in NYC, with darker areas corresponding to higher case rates (range 645-4,587 per 100,000 residents) Neighborhoods are defined as modified zip code tabulation areas. Data are from July 31, 2020 but the disparities were evident before, and after this date. Data collection and analysis was carried out by the NYC DOHMH’s Surveillance and Epidemiology Branch, while the data were collated by the NYC DOHMH’s Integrated Data Team.

Indeed, place-based approaches are increasingly utilized by public health organizations to combat health inequity and as a key reinvestment strategy to address ongoing structural and institutional racism.^19–21^ A place-based approach centers the unique determinants of health in a neighborhood, such as the quality of local housing or specific barriers to accessing healthcare. Similarly, a place-based approach leverages the unique resources and assets of a neighborhood, such as shared spaces in houses of worship, existing networks for health promotion, or the participation of trusted leaders. It is not difficult to anticipate the utility of a similar approach in public health emergency response, where disaster impacts often have inherent geographic attributes and where existing neighborhood level inequities compound those impacts.^22–24^

Place-based approaches have been implemented successfully in disaster preparedness and recovery phases^25,26^; however, translation of the place-based approach to the context of disaster response has not been straightforward. A significant limiting factor is the ability to collect and analyze data, particularly qualitative data, that are both directly relevant to evolving Incident Command System (ICS) objectives and operations, and are also timely and neighborhood-specific. This paper describes the design and implementation of the Community Assessment to Inform Rapid Response (CAIRR), a process for rapid collection and analysis of neighborhood-specific, objective-focused, qualitative data to inform tailored response operations, and its contribution to the NYC DOHMH’s novel *hyperlocal* approach, a rapid and time-limited place-based approach to disaster response, in the COVID-19 emergency. The aim of this paper is to share the NYC DOHMH’s experience and methods so that other jurisdictions seeking to implement a more equitable response can adapt the CAIRR for their own hyperlocal responses, not only to COVID-19, but also in future disasters.

## CAIRR development and initial implementation

### Overview

In the Spring of 2020, the NYC DOHMH’s ICS added a high priority incident objective to its COVID-19 emergency response: to stem transmission of SARS-CoV-2 in neighborhoods disproportionately impacted by COVID-19 through a hyperlocal approach. Recognizing the role of racial and other structural inequities in the geographic disparity of COVID-19 impact in NYC, 4 Neighborhood Response Teams (NRTs) were mobilized under the ICS Equity Officer as geographic divisions corresponding to 4 NYC boroughs (geographic units similar to counties) to achieve this objective. NRTs were responsible for coordinating operations in selected neighborhoods within their respective boroughs. Each NRT included operations, community engagement, data, and support staff.

This new hyperlocal approach built on prior emergency response efforts by NYC during Zika virus and measles outbreaks to leverage community partners’ input in response development. Conceptually, the hyperlocal approach drew heavily from the NYC DOHMH’s decades of place-based work to address health inequities in conditions such as HIV, maternal health, and chronic disease in neighborhoods most impacted by structural racism and disinvestment, exemplified by the establishment of NYC DOHMH’s Bureaus of Neighborhood Health (BNH) in 2003.^27^ Indeed, based on observations by the BNH early in the pandemic, the NRTs recognized the need for nuanced, neighborhood-specific operations that both leveraged existing community assets and addressed the specific barriers faced by these communities, but lacked neighborhood-specific contextual data to drive the design and implementation of such operations. At the same time, the demand for a response in the most impacted neighborhoods was urgent, requiring rapidity, scalability, flexibility in data (specifically qualitative data) collection, analysis, and operational design.

The CAIRR was created to satisfy each of these potentially conflicting requirements. To facilitate the rapid translation of qualitative data into operations, the NRTs first identified an existing operational framework already in use across the NYC DOHMH’s ICS, comprising 4 operational strategies which comprehensively encompassed all NYC DOHMH operations to stem transmission of SARS-CoV-2. These strategies were: *testing, source control, time, and space* (see definitions of *strategies* in Table 1). Second, the NRTs developed a simple qualitative tool based on this ‘*4 strategy’* framework, allowing the NRTs to sort and categorize data according to each *strategy* at the time of collection. Given the urgency of the objective and safety considerations related to COVID-19, rather than in-person field data collection, selected community partners who maintained regular communication with residents in the neighborhoods of interest were interviewed by phone. These interviews served as a proxy for direct field data collection from community residents, consistent with previously described rapid assessment processes.^28^ Finally, the use of the 4 *strategy* operational framework as an initial coding frame facilitated the rapid analysis and translation of collected data within each *strategy*, in order to directly inform corresponding operations.

**Table 1.** Key themes by operational s*trategy* to stem SARS-CoV-2 transmission

*Strategy*	*Key themes examples*	*Tailored operations examples*
Testing *Willingness and ability of individuals and communities to undergo COVID-19 testing and participate in contact tracing operations*	• Concern about testing cost • Fear of immigration enforcement at testing site • Not being able to find testing locations • Language access • Fear of entering government buildings	• Stand up new, free testing sites in Houses of Worship • Local organizations shared messages across social media that immigration status will not be asked ahead of operations. • Staff testing sites with interpreters speaking the most commonly encountered languages in the neighborhood
Source Control *Adherence to hand washing, wearing face coverings, and other individual behaviors to prevent transmission*	• Prohibitive price of PPE, price gouging for masks and sanitizer • Essential workers required to purchase their own required PPE • Lack of available PPE	• Distribute masks and hand sanitizer to local residents in areas of high foot traffic • Provide PPE to trusted local organizations for distribution • Coordinate with NYC enforcement agencies to facilitate anonymous reporting of price gouging • Share resources on employer obligations to provide PPE and mechanisms for redress with essential workers
Time *Minimizing exposure between individuals; this factor is impacted by crowded housing and ability to isolate*	• Multiple families share single apartment making it difficult to isolate in the home • Caregiving responsibilities prevent quarantining or isolating outside the home • Persons unaware of NYC hoteling program	• Station resource navigators at neighborhood rapid testing sites to connect persons in need to hotels and other support services • Share information about hoteling program, including accommodations for caregivers • Co-host webinars with local elected leadership specifically addressing neighborhood concerns
Space *Minimizing close contact between individuals in public spaces; this factor could be impacted by crowded workplaces or attending mass gatherings*	• Challenges to social distancing in public spaces • Customers don’t follow the rules in essential public spaces like grocery stores • Business owners don’t know how to encourage social distancing inside	• Distribute packets of floor stickers and posters to promote social distancing to local businesses • Recruit businesses into public-private partner groups to share strategies and information with other businesses in NYC • Canvas local businesses in high traffic areas to provide materials and education about SARS-CoV-2 transmission

The table illustrates some key themes that emerged from various neighborhoods and the tailored operations designed to address those particular themes. Themes were often common among 1 or more neighborhoods but the overal profile of themes from each neighborhood was unique; similarly, the suite of operations and operational details were unique for each neighborhood.

**Figure 2.**
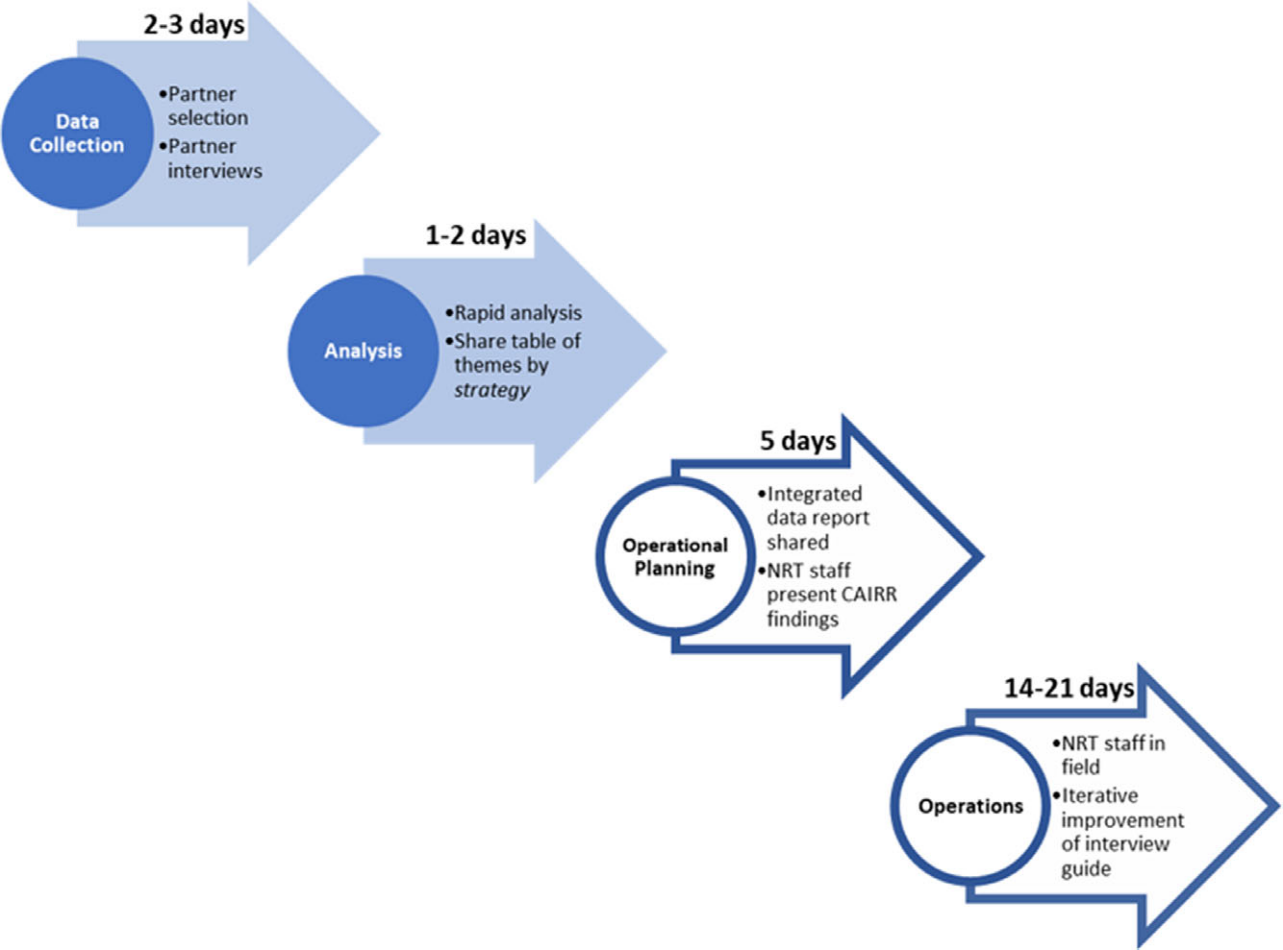
General timeline of hyperlocal response, including the CAIRR. The graphic illustrates a general timeline of the hyperlocal response in each neighborhood, beginning with neighborhood selection. The NRT assigned to a neighborhood’s borough was responsible for overseeing that borough. Shaded steps indicate the CAIRR, while those in outline were joint operational pieces. During the CAIRR’s early implementation, this timeline was executed once per neighborhood.

The CAIRR was integrated into the wider hyperlocal response, which operated across the ICS (Table 2) and engaged each selected neighborhood intensely for an operational period of several weeks (Figure 2) Upon selection of a neighborhood for hyperlocal response, the NRT corresponding to that neighborhood’s borough initiated the CAIRR and oversaw neighborhood-level joint operations. The entire CAIRR process spanned about 5 days. By day 6 after neighborhood selection, summary CAIRR data were shared with operational groups in a template-based report and through integration of an NRT representative into joint operational planning processes. Tailored neighborhood operations began around day 10 and lasted for a period of approximately 2-3 weeks. NRT staff led and participated in joint tailored operations based on CAIRR findings and other neighborhood-level data (see Table 1 for examples of findings and corresponding operations). The CAIRR was performed once for each neighborhood during this period of early implementation.

**Table 2.** Groups involved in the early implementation of the hyperlocal response

Response Group	ICS Organization	General Roles
Neighborhood Response Teams*	Divisions under Equity Officer	Oversight of hyperlocal response, CAIRR, Community and private partner engagement, Testing site operations, Resource navigation
Citywide Health Emergency Field Operations*	Branch under Unified Operations Section, Clinical Group	Testing site operations
Community Partner Engagement*	Unit under Public Information Officer	CAIRR, Community engagement, Testing site operations, Resource navigation
Healthcare Systems Support*	Branch under Operations Section, Clinical Group	Healthcare provider engagement
Integrated Data Team*	Branch under Science Section	Data integration across Response Groups, Demographic and GIS analysis
Laboratory*	Branch under Unified Operations Section, Clinical Group	Testing site operations
Liaison Officer	Liaison Officer	Coordination with NYC Agencies
Logistics*	Logistics Section	Resource mobilization
Mental Health*	Branch under Unified Operations Section, Clinical Group	Testing site operations
Planning Section*	Section	Coordination within DOHMH ICS, Forward planning
Public Information Officer*	Public Information Officer	Language interpretation and translation, Speaking events, Production of materials (flyers, posters, etc.)
Safety Officer*	Safety Officer	Testing site operations
Surveillance & Epidemiology	Branch under Unified Operations Section, Clinical Group	Surveillance, Epidemiological data analyses
NYC Health & Hospitals	External to NYC DOHMH	Testing site operations, Resource navigation
NYC Commission on Human Rights	External to NYC DOHMH	Support redress for price gouging and clarify employer obligations
NYC Department for the Aging	External to NYC DOHMH	Support engagement in Senior Centers
NYC Housing Authority	External to NYC DOHMH	Support testing sites in Section 8 housing
NYC Mayor’s Office of Immigration Affairs	External to NYC DOHMH	Amplify messaging to immigrant groups
NYC Small Business Services	External to NYC DOHMH	Amplify messaging to private partners
NYC Office of Emergency Management	External to NYC DOHMH	Amplify messaging to community and private partner networks, food pantries

Under NYC’s Citywide Incident Management System, a local adaptation of the National Incident Management System, the NYC DOHMH is a primary agency in a public health emergency, sharing unified command with the Fire Department of New York and the NYC Police Department.^58^ This phase of the hyperlocal response focused on NYC DOHMH and NYC Health & Hospitals’ activities, though other NYC Agencies provided support. Response groups marked with an asterisk (*) attended joint operational planning sessions.

### Neighborhood selection

Assignment of neighborhood priority for hyperlocal response, including the CAIRR, was determined by a steering committee comprised of the Incident Commander, the Equity Officer, and technical experts, and was based on multiple factors including historical disinvestment, assessment of social vulnerability, and the emergence of concerning COVID-19 epidemiological data such as SaTScan signals^29^ or a relative increase in neighborhood percent positivity.^30^ Modified zip code tabulation area boundaries were used to delineate neighborhoods in order to align with other emergency response operations.^31^

### Interview guide

A structured interview guide was developed based on the *4 strategy* operational framework to stem SARS-CoV-2 transmission (*testing, source control, space, and time*). The guide consisted of open-ended questions focused on the needs, barriers, assets, and experiences community partners identified from resident interactions corresponding to each of the 4 *strategies* (see supplementary materials for an early iteration of the interview guide). To avoid a 1-sided interaction, interviewers also offered resources to partners, such as support finding nearby testing sites, webinars by NYC DOHMH experts, or flyers with locally relevant information. Development of the interview guide was an iterative process, so that questions evolved based on previous lessons and observations, existing neighborhood level data, and emerging COVID-19 concerns.

### Community partner selection

Partners were identified using a number of sources, including established NYC DOHMH relationships, suggestions by staff with local knowledge, snowball selection via personal and professional networks, and existing partner databases and directories.

Since community partners acted as a proxy for direct data collection from neighborhood residents, only a subset of identified partners was selected strategically to reflect a variety of local organizations maintaining regular communication with a cross-section of neighborhood residents, such as community-based organizations, resident associations for public housing, faith-based organizations, schools, community boards, food pantries, and healthcare providers. An organization’s catchment population was also considered, and efforts were made to reach organizations serving specific priority populations such as seniors, immigrants, and LGBTQ+ individuals. Selected partners were listed on a shared spreadsheet where staff conducting phone interviews tracked all attempts and interactions.

### Data collection

Interviews were conducted in English or Spanish and lasted between 15 and 30 minutes. All interviews were conducted by NYC DOHMH staff, including data analysts and operations staff, with experience in community engagement, and/or qualitative data collection. Additionally, all interviewers received a brief technical training. In most cases, interviewers conducted follow-up with organizations to provide COVID-19 related resources or share updated information. All interviews were typically completed over 2-3 days, accounting for delays in contacting partners due to lockdowns,^32^ and transition to remote work across NYC. Interview responses were recorded in an online Alchemer survey form and downloaded into Microsoft Excel (Microsoft Corp, Redmond, Washington, USA) for analysis.

Early experience revealed that with well trained staff, thematic saturation was achieved with 10 or fewer completed partner interviews per neighborhood. Not surprisingly, organizations with strong prior relationships with the NYC DOHMH were more likely to engage with the NRTs, though interactions with new partners often led to ongoing relationships that continued after the hyperlocal response was complete.

### Rapid qualitative analysis

Analysis of partner interviews was conducted separately for each neighborhood. Responses were initially coded deductively using the 4 *strategy* operational framework, then inductively within each *strategy* utilizing the matrix analysis method, with the interview questions serving as the main domains, to produce 4 sets of key themes. This allowed for a quick understanding of the major findings, and could be completed by persons with minimal qualitative training.^33^ For each neighborhood, key themes were consolidated into a single table according to *strategy* and incorporated into a template report together with epidemiological and other quantitative data, neighborhood demographics, geographic information including mobility analyses, and a social vulnerability score. Reports were produced by the NYC DOHMH ICS Integrated Data Team and shared with operational groups from across the ICS by day 6 after the selection of a neighborhood for hyperlocal operations, ahead of joint operational planning sessions. When deemed relevant, more granular data were also shared internally with staff responsible for related field operations.

Coding, analysis of findings, and report writing were typically completed within 1 to 2 days immediately following completion of interviews. This time could be further reduced by beginning the process of coding and analysis of findings simultaneously with data collection.

### Operationalization

General operational plans were tailored to meet neighborhood characteristics during joint operational planning sessions that included representatives from involved operations (see Table 2 for a list of groups involved in hyperlocal operations). Operational leads reviewed CAIRR findings as part of an integrated data report ahead of the meeting, and an NRT representative participated in operational planning to support the application of relevant CAIRR findings, and to define NRT-led operations. The entire operational planning process, from the first joint operational planning session to the deployment of field staff, spanned approximately 5 days, and executions of operations spanned approximately 2-3 weeks (Table 1 illustrates examples of operations tailored to CAIRR findings).

## Assessment of success

Assessment of operational success varied across neighborhoods based on identified needs; for example, hyperlocal response in 1 neighborhood with low testing rates was deemed successful when that neighborhood’s SARS-CoV-2 testing rates rose to meet the citywide average, while response in another neighborhood with a SaTScan cluster was considered successful when the cluster was no longer detectable at the close of operations. Some outcomes, however, were difficult to quantify in the short term, such as development of new partnerships in a neighborhood previously distrustful of NYC DOHMH messaging, or correction of locally relevant misinformation through tailored webinars (see Table 1). The NYC DOHMH’s hyperlocal response would likely have benefited from a universal evaluation framework that matched the rapidity, scalability, and flexibility of the CAIRR.

## Discussion

### A place-based approach to ICS response

Geographic disparities in the impact of public health emergencies are not unique to COVID-19, nor are they limited to NYC. These disparities are explained through *social vulnerability*, a concept which encompasses downstream impacts of structural racism and other structural inequities (e.g., housing stability, health insurance access, and resiliency of the built environment), and can be estimated at the neighborhood level.^34–36^ Simultaneously, people living in highly impacted communities are often the biggest asset to disaster response and recovery, serving as both the ‘first and last responders’ in an emergency.^37,38^

Given that the geographic disparities encountered in public health emergencies have structural roots, it is not surprising that similar health disparities persist outside of an emergency context.^23,39^ In public health, social vulnerability and community resiliency can be approximated by a neighborhood’s *social determinants of health*, defined as the conditions in the environment where people are born, live, learn, work, play, worship, and age, that affect a wide range of health and quality-of-life outcomes and risks.^40^ The NYC DOHMH has a long history of place-based work, strengthened by renewed investment in neighborhoods with the development of the BNH teams in 2003.^41^

Disparities in COVID-19 outcomes stem from many of the social determinants of health addressed through the BNH’s place-based approach, but the scale of the pandemic required translation of the BNH’s long-term work to an emergency context.^42–47^ The BNH’s place-based approach leverages deep, long-term partnerships to attack neighborhood-level health inequities at their roots.^27,41^ Indeed, the BNH’s interventions early in the pandemic focused on addressing neighborhoods’ social determinants of health, such as food and housing insecurity, in addition to slowing SARS-CoV-2 transmission. However, given the urgency created by inequities in COVID-19 morbidity and mortality, the NRTs were mobilized for a much narrower and more time-bound task: the rapid execution of a suite of response operations to ensure a timely, specific response to concerning trends in SARS-CoV-2 transmission in neighborhoods impacted by racism and other structural inequities, or as the NYC DOHMH termed it, a *hyperlocal approach*.

### A common operating picture at the hyperlocal level

The CAIRR was fundamental to the adaptation of the NYC DOHMH’s place-based approach to a hyperlocal response under the ICS. Consequences of COVID-19 in a neighborhood are uniquely amplified by its particular barriers to stemming transmission and minimized by its particular assets; qualitative data about those barriers and assets were essential to developing tailored operations.

The use of neighborhoods or ‘small geographic areas’ for operational scale has a clear benefit to operations. Operations covering a smaller area can be adapted much more quickly and with fewer resources relative to citywide operations if accurate and timely data at the neighborhood level are available. However, measurement of accurate and precise quantitative data at the level of a small geographic area can be complex, as conclusions based on small datasets are more subject to errors created by missing data or non-representative sampling of data.^48^ Statistical data, epidemiological data, and other data such as mobility estimates are all critical to operations, but can be difficult to interpret at the neighborhood level without context. As such, the NYC DOHMH used epidemiologic data about SARS-CoV-2 transmission and outcomes, geographic and mobility data about high traffic locations, and demographic data about baseline health, race, and healthcare access to complement qualitative data, rather than using such data independently to develop hyperlocal operations.

Qualitative data collected and analyzed through the CAIRR provided nuanced, operationally relevant context in a timely fashion, acting as a narrative glue for disparate data from different ICS sections and branches collected through different methods. The CAIRR made a common operating picture possible for the hyperlocal response, allowing ICS leadership from the Incident Commander to Operational Leads to access a single, template-based report for situational awareness and informed decision-making, reflecting data collected in near-real time.

Different jurisdictions may find different geographic units more appropriate for the CAIRR and for a hyperlocal response. A few competing considerations are important to selecting a geographic unit. Administrative units are often selected because of data availability; response is not the ideal time to revamp statistical calculations to reshape epidemiological data.^49^ However, administrative boundaries may not reflect critical elements of a neighborhood such as community networks^50,51^ or common demographic characteristics.^52^ Conversely, definitions of neighborhoods based on common demographics or community networks can be difficult operationally, requiring staff and partners in the field to navigate irregular operational boundaries. Another option is a grid-based system which does not take into account neighborhood dynamics or existing administrative boundaries, but simplifies turf-cutting and interoperability between jurisdictions.^53^ Although the CAIRR can be adapted quickly if rough geographical information about partner catchment areas are available, units based on neighborhood dynamics or a grid system will require work in the preparedness phase to update quantitative and epidemiological analytic systems and to familiarize partners with new operational boundaries.

The NYC DOHMH used zip codes (defined as modified zip code tabulation areas^31^) as its common geographical unit, sometimes extending operations to 2 or 3 adjacent zip codes depending on total area, population, and neighborhood context. Although zip codes in NYC often intersect haphazardly with community definitions of neighborhoods, they were a common enough boundary that community members, field staff, and groups outside of the jurisdiction had a reasonable understanding of the rough boundaries. Perhaps most critically, other ICS sections and branches had already established data systems for demographic, epidemiological, mobility, and other data at the zip code level. Still, use of zip codes did create confusion among external partners and community members about why 1 part of a community-defined neighborhood encompassing multiple zip codes was receiving attention while another was not; additionally, given that networks often extend across zip code lines, it is quite possible that responses may have missed drivers of transmission whose origin was just over the zip code border.

### Rapidity and scalability in data collection

A salient feature of the CAIRR is the rapidity at which qualitative data are collected and analyzed; strategic partner selection is key to this rapidity. Through selection of well-connected and well-informed individuals from organizations that worked with a diversity of populations in each neighborhood, the NRTs were able to gather critical information on community perspectives and behavior, attaining thematic saturation after only a small number of interviews (see Table 1). Of note, while partner selection was easier in neighborhoods where the NYC DOHMH had strong pre-existing relationships, the model was still successful in other neighborhoods, although it required more effort to connect with new partners who were both informed and willing to participate. Partners also had an opportunity during the interview to request information and resources to support their COVID-19 prevention efforts, including webinars, literature, and data. This created a symbiotic relationship that went beyond the initial data collection process, and in some cases developed into an ongoing partnership with new partners, or deepened relationships with existing partners.

By design, the CAIRR did not attempt to obtain a representative sample of interview participants, but rather focused on strategic selection of key partners in the interest of rapid provision of appropriate services to those residents whose immediate, unmet needs the NYC DOHMH could gather specific data about. The CAIRR was intended to complement rather than replace more comprehensive research methods which may better assess the needs and assets of hard to reach communities but which may require more time to complete.^54,55^ Toolkits for rapid needs assessments do exist to achieve statistically representative qualitative data at the household level, particularly the Centers for Disease Control and Prevention’s (CDC) Community Assessment for Public Health Emergency Response (CASPER).^56^ However, at the time the NYC DOHMH initiated the hyperlocal response, methods such as CASPER were not feasible. In particular, the CASPER requires field data collection; early in the COVID-19 response, staff were only deployed in-person if absolutely necessary due to SARS-CoV-2 transmission risk. Additionally, household-level sampling necessarily requires interviews with a random selection of community members. Trust in government was often very low amongst residents of neighborhoods experiencing high levels of SARS-CoV-2 transmission, while community partners such as faith-based organizations or mutual aid networks retained the confidence of their communities.

Other jurisdictions experiencing similar situational challenges or without the resources or the time to do comprehensive qualitative assessments can modify the CAIRR to enable rapid, hyperlocal, objective-focused emergency response applicable to their localities by leveraging the expertise of locally active community organizations.

### An operational framework for analysis

The task of translating qualitative data to operations can be difficult and time consuming. The CAIRR, however, was designed with public health emergency operations in mind, particularly the primary objective of stemming SARS-CoV-2 transmission through a hyperlocal approach. Rather than explore root causes of disparities in SARS-CoV-2 transmission, the CAIRR adopted a simple, high-level operational framework for addressing transmission that was already in use in multiple ICS operations as an initial coding frame. Analysis did not begin with the question, ‘why is SARS-CoV-2 transmission different in some NYC neighborhoods than others?’ Instead, the CAIRR aimed to gather just enough data to deploy a field operation that met the specific needs of the neighborhood.

This approach facilitated rapid qualitative analysis and translation to objective-focused operations. Starting with the operational *4 strategy* framework allowed analysts to begin with a deductive approach, guaranteeing the emergence of themes that could broadly inform operations at the neighborhood level and additionally saving time relative to approaching the full dataset inductively. Analysts could then use a secondary inductive approach leveraging matrix analysis methods within each of the *strategies* to qualify more granular neighborhood data (see Table 1), A similar process in which a high-level coding frame is mapped to potential operations prior to data collection has since been adopted in other emergency response toolkits, such as the CDC’s Vaccine Confidence Rapid Community Assessment.^57^

Additionally, since the structured data collection and matrix analysis methods facilitated by the operational framework simplify an otherwise laborious process, the CAIRR can be adapted and implemented even in jurisdictions with limited qualitative research capacity. At the NYC DOHMH, NRT staff who brought other critical assets to the work (i.e., prior community engagement experience, fluency in relevant languages, and residence or lived experience in a community of focus) participated successfully as data collectors after just-in-time training in qualitative data collection. Similarly, the analytic team was limited to a small number of researchers with technical skills in Microsoft Excel (Microsoft Corp., Redmond, Washington, USA) who received just-in-time training on the analysis framework. This approach was not only sufficient to conduct the CAIRR, but also enriched its findings by leveraging the unique skills and abilities of individual team members. Furthermore, NRT staff who acquired new qualitative skills during the hyperlocal response simultaneously added to their own professional development and helped to increase NYC DOHMH’s overall qualitative research capacity, both for disaster response and for public health programming outside of an-emergency response context.

The value of the operational framework transcends its analytic speed and simplicity. Although community engagement response operations are often based on qualitative impressions of community needs, qualitative data without operational context can be difficult for leadership to respond to. By starting with an operational framework already familiar to leadership across the ICS, the NRTs were able to elevate timely data collected from impacted communities to leadership in language that was actionable and understandable. Additionally, the operational framework created a mechanism to “match” the community input represented in those data to existing operations categorized in the same *strategy*, thereby translating that input into governmental action within a few days (Table 1).

One should note that, significant adaptation of the operational framework in the midst of a response may require some forward planning, particularly around integration of CAIRR findings into citywide operations. For example, when themes around a potential COVID-19 vaccine emerged far ahead of any vaccine authorization, the CAIRR’s *4 strategy* framework was easily expanded to include a fifth *vaccine strategy* for the purpose of data collection and analysis; however, the full benefits of these analyses could not be realized until they were integrated with neighborhood-level operational planning around vaccines.

Rapidity, flexibility, and other strengths of the CAIRR are not particular to the COVID-19 emergency nor to the specific operational framework used by the NYC DOHMH’s ICS. As long as a framework is consistent across the breadth of operational sections and branches in a response, jurisdictions can tailor the CAIRR to meet the demands of their individual objectives and associated operations.

## Conclusion

Neighborhood-level differences in social vulnerability and community resilience create neighborhood-level differences in disaster impact, which demands a neighborhood-level response. Place-based approaches in public health acknowledge that the outcomes of structural racism and other inequities result in geographic differences within a jurisdiction. However, translation of the place-based approach to disaster response has previously been limited by the ability to collect timely, relevant qualitative data at the neighborhood level. In the face of mounting inequities in COVID-19 impact between neighborhoods, the NYC DOHMH developed the CAIRR, and consequently demonstrated the feasibility of a hyperlocal response to public health emergencies. The CAIRR produces the qualitative analyses necessary to tie together diverse emergency operations, contextualize complex datasets, and provide critical decision support to ICS leadership. While the CAIRR was not designed to produce comprehensive analyses at the neighborhood level, it is a scalable and flexible process that can be adapted for different jurisdictions facing different disasters and with different resource limitations. Jurisdictions seeking to implement a more equitable response can modify the CAIRR for their own hyperlocal responses to COVID-19 and future disasters.

## Supplementary material

For supplementary material accompanying this paper visit https://doi.org/10.1017/dmp.2022.135. click here to view supplementary material
