# Supplementary material for: The Community Assessment to Inform Rapid Response (CAIRR): A Novel Qualitative Data Collection and Analytic Process to Facilitate Hyperlocal COVID-19 Emergency Response Operations in New York City
Source: Disaster Med Public Health Prep. 2022 May 30:1–10. doi: 10.1017/dmp.2022.135 (PMC9300969; doi:10.1017/dmp.2022.135)
Supplement: Supplementary file 1 [file S1935789322001355sup001.pdf]

INTERVIEW GUIDE

READ THE FOLLOWING:
Hello. My name is ___________ and I work for the [Bronx Neighborhood Response Team], part of the NYC Department of Health and Mental Hygiene. I'm calling to check-in with you about the Coronavirus crisis also known as COVID-19. How are you dealing with the crisis? In an effort to help end transmission of the virus, the Department of Health is reaching out to various community stakeholders, like yourself, who have regular contact with community members to help support you with information and accessing resources, particularly related to key factors that we know affect transmission. We would also like to better understand how the community feels about various measures that are needed to help end transmission, and we're hoping you could help us answer some of these questions. In particular, today I am calling to talk with you about [neighborhood] which we are considering to be [zip codes]. Although you may work in many neighborhoods across the city, anything you can share specifically about this neighborhood would be especially helpful to us.

Is this a good time to speak with you? *(If yes, continue. If no, ask for a better time.)*

Are you (or your organization) in contact with the community during this crisis and hearing about their concerns? *(If yes, continue.)*

(If no) Please refer me to someone in your organization who in your opinion can best answer questions related to community concerns about COVID-19.

TESTING
We recommend that all New Yorkers should get tested for Coronavirus now, whether or not they have symptoms or are at increased risk. Because of this, we are reaching out to you as a partner of DOHMH to help us spread accurate information about the test and trace program. First, I want to share a few important facts with you:

- We recommend that all New Yorkers make an appointment to get tested now. If your first COVID test is negative, you should get tested again if you
  - are concerned about possible exposure
  - have spent time in a large crowd
  - have had exposure to someone with confirmed or possible COVID-19
  - have symptoms
  - work in a congregate setting like a nursing home or shelter and are planning to visit someone at high risk for severe COVID-19 illness
- Test results are completely confidential, and results will be returned to the patient usually within about 48 hours.
- The test will be done as a nasal swab, oral swab, or using a saliva sample.
- You do NOT need insurance to get a test, although some testing sites may ask for insurance information as some insurance companies are paying for tests, and

for information purposes. If someone does needs help signing up for insurance, they can call 311.

- There are hundreds of sites across the city. The nearest testing site to your organization's location is: [FILL IN ADDRESS AND INCLUDE INFORMATION ABOUT APPOINTMENTS AT THAT SITE.] You can reference this website to help you find information about this or any other testing site: https://www1.nyc.gov/site/coronavirus/get-tested/covid-19-testing.page

1) Do you have any questions about this information that I can help to clarify?
2) What are some of the challenges that you have heard from community members that prevent them from getting tested for COVID-19? *(PROBE: Perceptions/held beliefs/obstacles/movement)*
    - ☐ Knowledge of testing locations
    - ☐ Cost of tests
    - ☐ Fear due to their own or others' immigration status
    - ☐ Other: ___________________________________

Specify any detailed information on the response above here: ______________________

SPACE

We know that minimizing close contact between individuals in public spaces can help to limit the spread of COVID-19. For the reason, we strongly encourage everyone to remain 6 feet apart from people who do not live in their home, even other family members or close friends, at all times. We refer to this as "social distancing".

3) What have you been hearing from community members about social distancing in public, including in parks, grocery stores, public transportation, faith-based services, etc.?

____
____
____

4) What can the Health Department do to help community members (and your organization) follow social distancing protocols to prevent spread of COVID-19? *(Such as, developing distancing guidelines, providing you with FAQ documents, develop/send targeted text messaging to this zip code, etc.)*

____
____
____

TIME

Minimizing prolonged close contact between individual helps limit the spread of COVID-19, but it is made more difficult by crowded housing and the ability to isolate if someone tests positive or suspects they might be positive for COVID-19.

5) What have you heard from community members about their ability to stay apart from household members if they are sick?

____

____

____

6) Have you heard of any residential buildings in the neighborhood that are not being properly sanitized or have high rates of COVID-19 transmission?

____

____

____

SOURCE CONTROL

As you know, the Health Department has been promoting important messages about what individuals can do to minimize their risk, including hand washing and wearing face coverings outside of the home.

7) What barriers have you heard community members speak of with regards to using face coverings and hand sanitizer?

____

____

____

HOW CAN WE WORK TOGETHER

In addition to wanting to help spread accurate information about testing, we know that residents and partners have many questions about COVID-19 in general and how it spreads. We want to ensure that NYC residents are receiving the most up-to-date, credible information about COVID. We are very interested in finding ways to share information with our partners, since we know you are a credible messenger in the community who can help to share important information.

8) Would you be interested in hosting a webinar or having a DOHMH facilitated webinar for your clients about COVID data and DOHMH response efforts to address the impact of the virus?
   - ☐ Yes
   - ☐ No

If yes, ask the partner for:

- Contact info:
- Date/Time:
- Topics they want covered: Covid Response/Info, Supply Chain/Materials Management, Personal Preparedness, Mental Health, Workforce Concerns, Social Distancing, Health Equity/Stigma, Congregate Shelter Guidance, Healthcare System Support, Community Preparedness/Resilience, Clinical Guidance, Healthcare Advocacy/Public Charge, Health Safety Guidance, Local/State/Federal Policy Education, Health Information Privacy/Security.
- Estimated # of ppl expected
- If they want a clinician or any other special requests

- Whether or not Press or Elected Officials are invited
- Language other than English needed?
- Do they have a video conferencing platform for the webinar? If yes, they should use that. If no, staff must indicate this in the initial survey gizmo speakers bureau form.

Immediately AFTER this call, you should enter the Speaker's Bureau request on behalf of the partner. [redacted]

9) Would you like us to add you to our mailing list to receive a weekly newsletter about COVID data and DOHMH response to address the impact of the virus:
   - ☐ Yes
   - ☐ No

If yes, let them know that they will be signed up for the newsletter and will begin receiving it within a few days.
If they want to sign up additional colleagues, you can share this link with them: [redacted]
For FBOs only, let them know that there is also a biweekly conference call they can choose to join:

> DOHMH is hosting a bi-weekly informational call for community and faith leaders with the latest updates on COVID-19. Come learn about the variety of services and resources offered by NYC government during this COVID-19 pandemic. Guest speakers will also discuss the City's 2020 Heat Plan and resources available for homeowners. This call is hosted by NYC Emergency Management Community Preparedness with experts from Department of Health & Mental Hygiene, Dept. of Housing & Urban Development (HUD), the Mayor's Office of Immigrant Affairs, Commission on Human Rights and Mayor's Office of Community Affairs. Ask partner if interested in being added to the listserv.

CLOSING

10) We are very aware that there are great disparities in the effects of COVID, and the city has prioritized an equitable response. Can you please me tell me which zip codes your organizations serves? *SELECT FROM CHECKLIST.*
11) Is your organization serving any specific populations during this time? *SELECT FROM CHECKLIST [immigrants, youth, older adults, LGBTQ+, …]*

Thank you for talking with me today. If you have additional needs that come up that we could help with, we can be reached at [718-XXX-XXXX or [redacted]@health.nyc.gov].

I can also email this information to you as a follow-up to this call. What is the best email address to reach you at? ______________________________
